# ‘I imagine teams!’ – exploring the potential of team-based long-term brain injury rehabilitation in North Norway

**DOI:** 10.1186/s12913-025-13894-0

**Published:** 2025-12-29

**Authors:** Morten Nikolaisen, Cathrine Arntzen, Marianne Eliassen, Lina Forslund, Hege K. Andreassen, Astrid Gramstad

**Affiliations:** 1 Department of Health and Care Sciences, Faculty of Health Sciences, UiT The Arctic University of Norway, 9037 Tromsø, Norway; 2 Division of Rehabilitation Services, University Hospital of North Norway, 9038 Tromsø, Norway; 3 Department of Health and Care Sciences Gjøvik, Faculty of Medicine and Health Sciences, NTNU, 2802 Gjøvik, Norway

**Keywords:** Brain injuries, Co-design, Healthcare teams, Interdisciplinary care, Rehabilitation, Rural areas

## Abstract

**Background:**

Acquired brain injury (ABI) is a leading cause of disability in adults worldwide, often resulting in long-term impairments that hinder community integration. While advancements in acute care have improved ABI survival rates, significant gaps remain in long-term follow-up. These gaps are exacerbated in rural areas, where dispersed populations, long distances, and limited access to rehabilitation professionals pose challenges. Although team-based approaches are crucial for effective ABI rehabilitation, these approaches have yet to be fully integrated into long-term care, thus highlighting the need for innovation. This study explores the potential of team-based organisation in improving long-term rehabilitation services for individuals with ABI in the rural context of North Norway.

**Methods:**

This study employed a collaborative knowledge generation framework inspired by Experience-based Co-design, wherein researchers work with stakeholders to align research efforts and service development. Data were generated during a full-day workshop during which two focus groups comprising health and welfare professionals (*n* = 10) and individuals with ABI (*n* = 3) explored the potential of team-based organisation in improving long-term ABI rehabilitation services in a rural context. The data were analysed using a predominantly inductive approach to thematic analysis.

**Results:**

Four key themes were identified: (1) establishing municipal ‘core teams’ at the primary healthcare level to strengthen local rehabilitation services; (2) enhancing care continuity through ongoing collaboration between the core teams and existing specialist ambulatory rehabilitation teams; (3) sharing the management of long-term rehabilitation trajectories between teams; and (4) using teams as platforms for professional learning and knowledge sharing. These themes informed the construction of a framework designed to guide the development and application of team-based long-term ABI rehabilitation.

**Conclusions:**

This study highlights the potential of team-based organisation in enhancing long-term rehabilitation services for individuals with ABI in rural contexts. Establishing core teams within municipalities and fostering ongoing collaboration between these teams and specialist healthcare teams can bridge service gaps to promote community integration and self-management. The proposed framework can guide the development and application of team-based long-term ABI rehabilitation in rural regions. Further research is needed to evaluate the framework’s real-world applicability and impact on rehabilitation outcomes, professional development, and overall service effectiveness.

**Supplementary Information:**

The online version contains supplementary material available at 10.1186/s12913-025-13894-0.

## Background

Acquired brain injury (ABI), which includes conditions such as stroke and traumatic brain injury, is a significant cause of disability in adults worldwide [1–5]. ABIs often result in various impairments that affect motor, sensory, cognitive, perceptual, and emotional functions, leading to long-lasting disabilities that hinder activities and community integration after hospital discharge [6–9]. Advances in acute medical care and subacute rehabilitation, such as thrombolysis [10, 11] and stroke units [12, 13], have improved survival rates and mitigated injury severity among individuals with ABI (iwABI).

However, while ABIs rightfully are given high priority in acute care, significant gaps remain in longer-term follow-up after hospital discharge [14–23]. These gaps are even more pronounced in rural areas, where healthcare delivery is limited by the low availability of local resources, inadequate access to specialised services, and long travel distances [24–26]. Consequently, iwABI in rural areas often lack professional support as they struggle to adapt to everyday life in the community, and this process can persist for several years [6–8, 27–30]. Therefore, it is necessary to develop comprehensive long-term rehabilitation services that align with current definitions of rehabilitation. In Norway, the recently expanded legal definition of rehabilitation includes adapting to new life circumstances, returning to valued roles, and being reintegrated into the community [31, 32]. These aspects are increasingly considered overarching goals in long-term rehabilitation following ABI [33–36].

### Norwegian healthcare services

The Norwegian healthcare system operates on a two-tier model. The specialist healthcare level includes hospitals and is organised into four regional entities governed by the Ministry of Health and Care Services. Primary healthcare is managed by local authorities at the municipal level. Although the overall healthcare policy is formulated by the government, the responsibility for long-term care provision, including rehabilitation after hospital discharge, is delegated to Norway’s 357 municipalities. Municipalities, which constitute the foundational level of public administration and local democracy in Norway, exercise considerable autonomy in shaping their own healthcare services. Despite Norway’s long-standing commitment to principles of universal access and tailored care [37, 38], the combination of strong local governance and many small municipalities has led to significant variation in the availability and organisation of rehabilitation services [39]. In North Norway (Box 1), these challenges are exacerbated by the region’s dispersed population, long travel distances, and harsh weather, thus creating a challenging context for healthcare delivery.

**Box 1:**
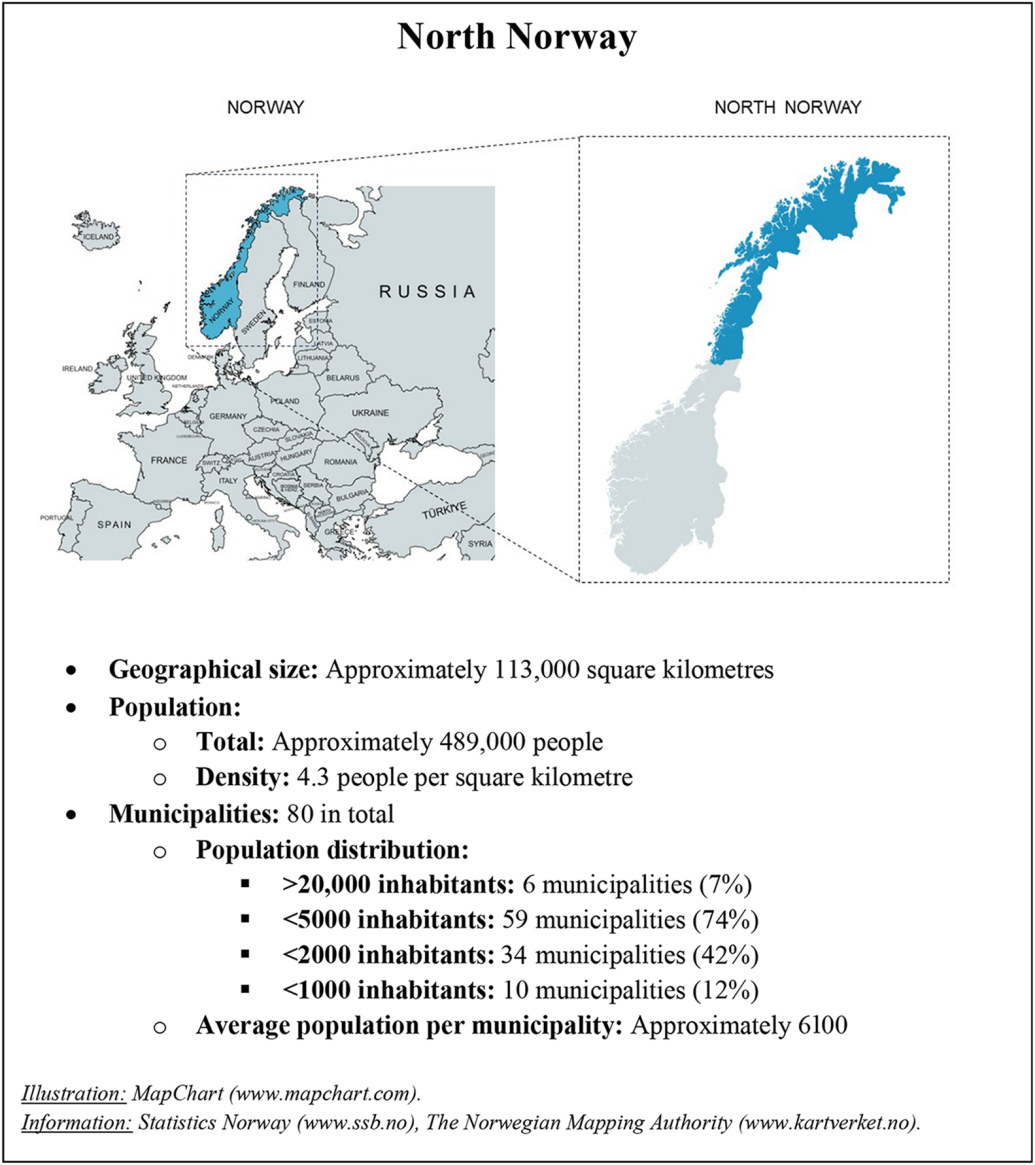
Geographic and demographic overview of North Norway

### ABI rehabilitation in North Norway

In line with trends in other Western countries, Norway has shifted towards shorter hospital stays and has transferred care responsibilities from specialist to primary healthcare [40–43]. Despite a substantial reduction in hospital stay duration, there has been no significant increase in municipal capacity or expertise in Norway [44, 45]. Within the field of rehabilitation, audits have revealed that activities at the specialist healthcare level have steadily declined, but no corresponding growth has been observed in municipal healthcare, thus leading to deficiencies in rehabilitation services after discharge to the community [45, 46]. Admittedly, over the past 15 years, Norwegian government authorities have demonstrated a persistent commitment to improving neurorehabilitation services through several reform initiatives involving both specialist healthcare and the municipalities [47–50]. However, Norway’s reform efforts have predominantly fallen short of producing substantial improvements in long-term rehabilitation services for iwABI, and significant gaps continue to exist between practice and the ideals reflected in policies [44–46].

To address these gaps, the specialist healthcare sector in North Norway established Ambulatory Rehabilitation Teams (ARTs) over two decades ago. These teams were created to enhance the presence of specialist healthcare across the region, thus demonstrating a commitment to improving long-term rehabilitation outcomes for iwABI irrespective of their place of residence. Currently, six ARTs, each comprising three to six members, cover geographical areas ranging from 1000 to 50,000 square kilometres. The mandate of these ARTs includes assisting iwABI as they transition to community living, coordinating care, and guiding municipal care providers.

Despite the presence of ARTs, recent studies focusing specifically on long-term ABI rehabilitation in North Norway have revealed significant shortcomings in professional support for cognitive impairments, psychosocial issues, the transition to everyday life, and community integration [51–55]. These research findings highlight a substantial misalignment between the long-term needs of iwABI and the current content and organisation of rehabilitation services in North Norway. This misalignment includes a lack of professional support in gaining insights into functional problems, identifying and expressing needs and goals, and navigating a complex and fragmented health and social care system [51–55].

### Team-based approaches to long-term ABI rehabilitation

Both the literature [51, 56–58] and Norwegian healthcare policies [42, 50, 59–62] emphasise the use of team-based organisation to address current challenges with rehabilitation service provision. Teamwork is generally considered essential for effective service delivery throughout the ABI rehabilitation trajectory because of its ability to provide a range of expertise in a coordinated manner while remaining centred on individual needs [56, 57, 63]. However, previous research has indicated that the availability of team-based ABI rehabilitation is limited after hospital discharge [64]. Existing team-based approaches to long-term ABI rehabilitation tend to fall short in terms of customisation, coordination, and proactive strategies, which are considered critical elements for successful team-based rehabilitation [17, 64, 65]. There is also a scarcity of literature on establishing and maintaining effective rehabilitation teams in primary healthcare, which reflects the overall neglect of team-based approaches to ABI rehabilitation after discharge to the community [66]. Furthermore, team-based approaches for long-term ABI rehabilitation in rural areas remain largely unexplored in the literature [67]. Collectively, these findings indicate a pressing need to develop new and innovative ways of delivering team-based, long-term ABI rehabilitation that is adapted to rural contexts.

### Study aim

This study aims to explore and discuss the potential of team-based organisation in enhancing long-term rehabilitation services for iwABI within the rural context of North Norway, drawing on insights from iwABI and professional service providers.

## Methods

### Study design

To support the development of a new and innovative approach to healthcare delivery, this study adopted a collaborative knowledge generation framework. Within this framework, researchers work with stakeholders to align research efforts with service development [68]. Specifically, the research design was inspired by the principles of Experience-based Co-design (EBCD), which emphasises the central role of service recipients’ experiences in driving improvement and innovation in healthcare [68–70]. This approach holds special relevance for research that aims to contribute to the ongoing development of healthcare services, as service recipients’ experiences and feedback are crucial for matching services to their needs. Given the unique challenges faced by iwABI in rural settings, EBCD offers a framework that ensures that these individuals’ voices are not just heard but are considered pivotal in research and service development.

**Fig. 1:**
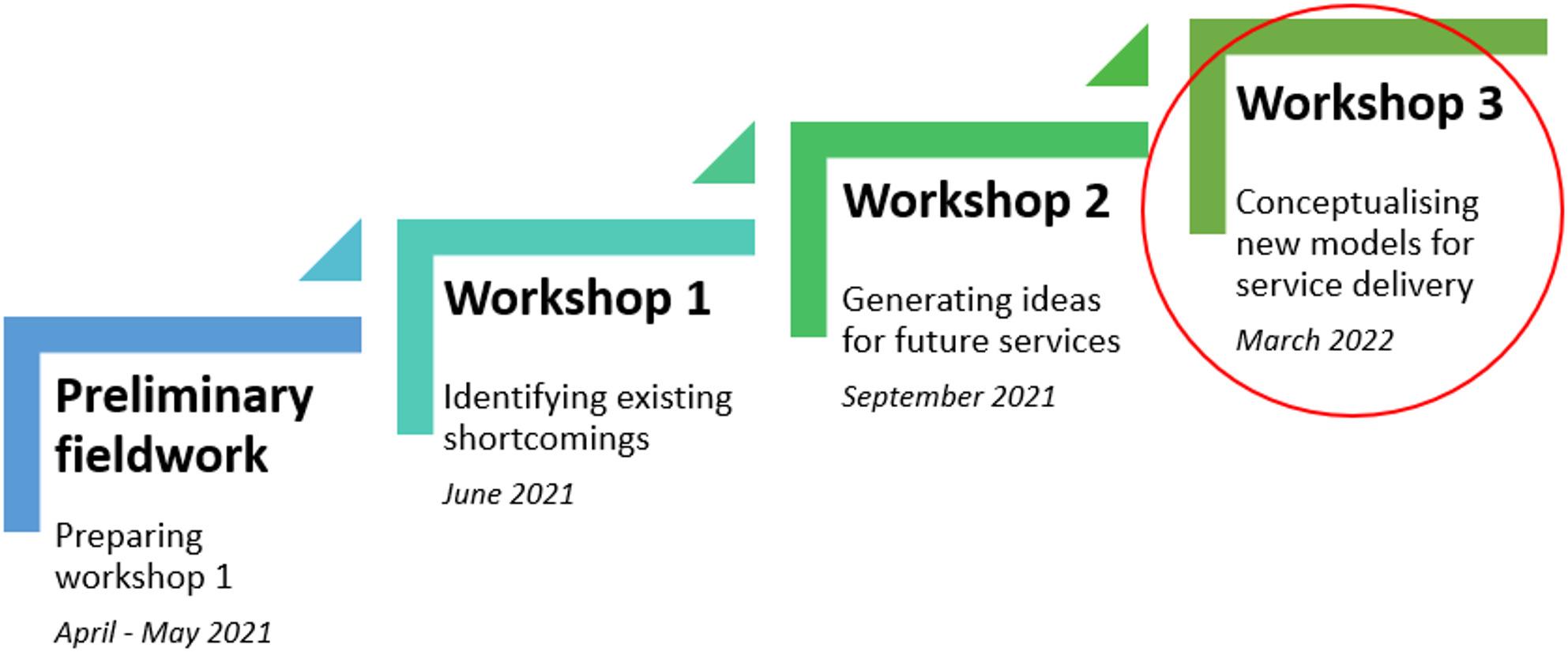
Outline of phases in the collaborative knowledge generation process of the project. This article reports findings from Workshop 3, circled in red

In this study, the EBCD-inspired approach is evident in the extensive involvement of iwABI and service providers as research participants. This was operationalised through initial preliminary fieldwork followed by three full-day workshops (Fig. 1). The fieldwork, which was conducted in North Norway by authors MN and LF, involved visits to home-dwelling iwABI, the office of a patient advocacy organisation, and rehabilitation service providers in both specialist and primary healthcare settings. The primary aim of the fieldwork was to explore the experiences of individuals involved in long-term ABI rehabilitation, with a particular focus on the interaction between iwABI and the healthcare system. Fieldwork data were not analysed in detail as part of this study but were essential in preparing and setting the stage for three subsequent full-day workshops. These workshops involved collaboration between iwABI, family caregivers, health and social care professionals, and researchers. The workshops progressed from identifying existing service shortcomings (Workshop 1) to generating ideas for future services (Workshop 2) and conceptualising new modes of service delivery (Workshop 3). This process has also been outlined in previous publications [71, 72].

**Fig. 2:**
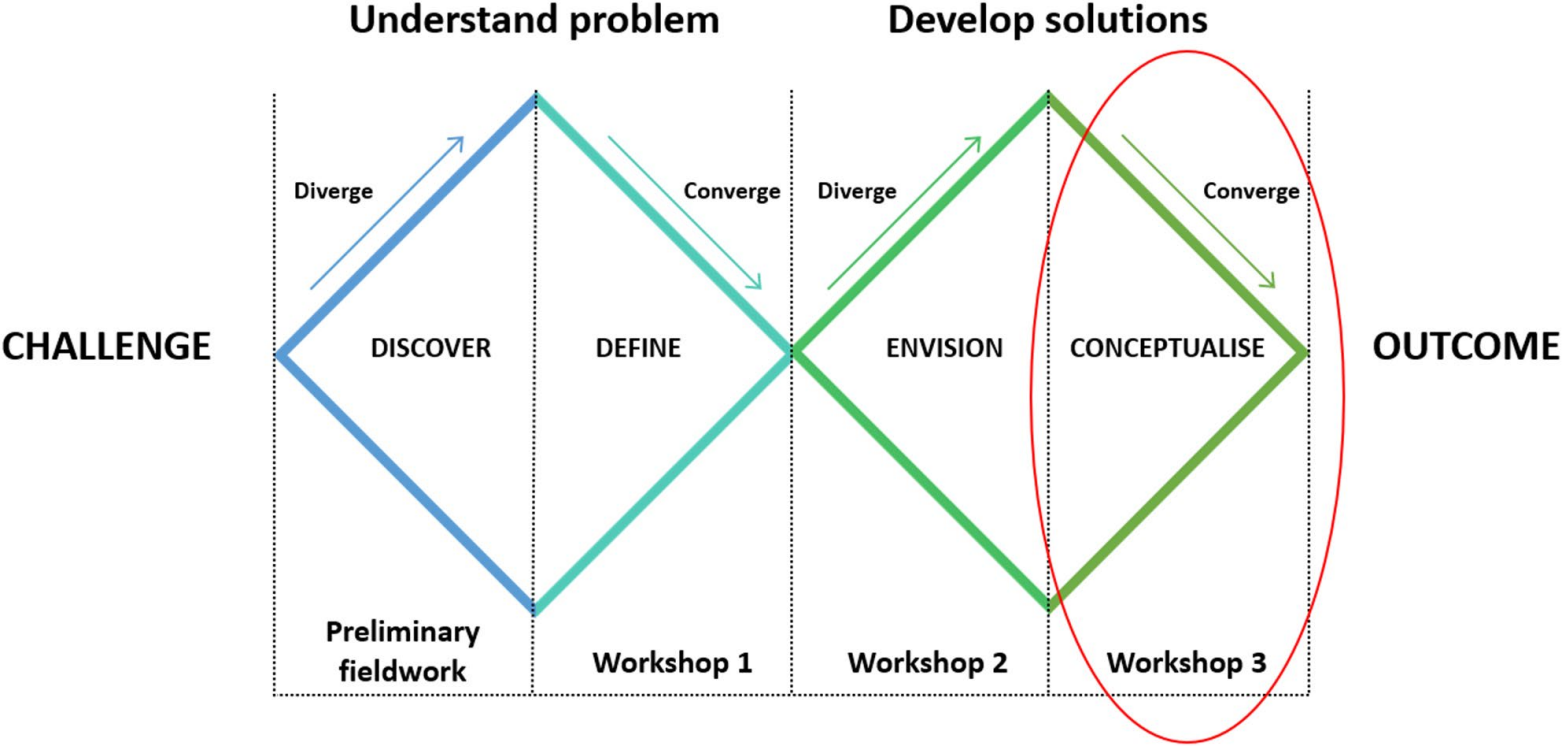
Outline of the design process phases of the project. The project phases outline is inspired by the Double Diamond as described by the Design Council (Design Council. The Double Diamond. A universally accepted depiction of the design process 2023 [cited 2023 October 30]. Available from: https://www.designcouncil.org.uk/our-resources/the-double-diamond/). This article reports findings from Workshop 3 (the conceptualisation phase), circled in red

Preliminary analysis of data from workshops 1 and 2 revealed that team-based service organisation was a common suggestion for enhancing long-term ABI rehabilitation in North Norway. However, the analysis also identified the need for a deeper exploration of the team concept in the context of long-term ABI rehabilitation in a rural region. Consequently, two focus groups were conducted to further explore and discuss the potential of team-based organisation during Workshop 3. This study focused on the analysis of data generated from these two focus groups, as well as their participation in a plenary session with all workshop participants during Workshop 3. Figure 1 provides an overview of the entire EBCD process. Figure 2 illustrates the distinct project phases structured according to the ‘Double Diamond’ model [73], which is a widely accepted representation of service design processes that was also used to communicate the project phases to research participants. In both these figures, Workshop 3 is circled in red.

### Participants

To ensure the relevance and depth of the data, we employed a purposeful sampling strategy to recruit workshop participants with diverse knowledge and experiences regarding rehabilitation services for iwABI in North Norway. Recruitment took place between February and May 2021 through formal agreements with relevant organisations, including two healthcare authorities (specialist healthcare), two municipalities, a private rehabilitation institution, the Norwegian Labour and Welfare Administration (Nav), and two patient advocacy organisations. In total, 27 participants took part in the workshop series, comprising 4 iwABI, 2 family caregivers, and 21 service providers. Participants were primarily recruited through the organisations, with managers or designated contacts assisting in identifying and inviting participants. The only exceptions were two participants with ABI and one family caregiver, who were recruited via service providers already included in the study.

For service providers, inclusion criteria required familiarity with service provision for iwABI in North Norway. Efforts were made to ensure a broad representation of professional backgrounds, geographic locations (e.g., large and small municipalities), and roles within the health and welfare service system (e.g., experience from various service settings). For participants with ABI, inclusion criteria required personal experience of sustaining an ABI and receiving rehabilitation services. For family caregivers, the inclusion criterion was experience in caring for a family member who met the inclusion criteria for participants with ABI.

Exclusion criteria included service providers without relevant experience in ABI rehabilitation in North Norway, iwABI or family caregivers whose experiences were unrelated to North Norway, and individuals who were unable to commit to attending the workshops. Additionally, iwABI who had severe cognitive or communication impairments that would prevent meaningful participation in the workshops were excluded.

The total number of workshop participants (*n* = 27) was considered appropriate to achieve the study’s aim of collaborative knowledge generation. It ensured that a diverse range of perspectives informed the discussions, while keeping the group size manageable within the practical constraints of the workshops. Furthermore, this number of participants was considered appropriate to facilitate collaboration over time and at each workshop. In the context of this study, fostering relationships among participants and creating a safe environment where they could freely express their ideas and opinions was vital, and could have been more challenging to achieve with a larger participant group.

13 of the 27 participants joined the two focus groups and discussed team-based approaches at Workshop 3. The two focus groups comprised iwABI (*n* = 3; 2 females, 1 male), healthcare professionals (HCPs) (*n* = 8; 7 females, 1 male), and staff from the Norwegian Labour and Welfare Administration (*n* = 2; both female). The time since injury for the iwABI varied from five to eight years. The health and social care professionals represented diverse organisational settings and varied experiences from different phases of the rehabilitation trajectory. Further participant details can be found in Table 1 (iwABI) and Table 2 (service providers).

**Table 1:** Characteristics of the participants with ABI

Focus group no.	Participant ID	Age	Time since brain injury	Size of home municipality
1	P1	40–49 years	5 years	< 5000 inhabitants
2	P2	50–59 years	8 years	< 10,000 inhabitants
2	P3	40–49 years	7 years	> 50,000 inhabitants

**Table 2:** Characteristics of the health and welfare professional participants

Focus group no.	Participant ID	Professional background	Organisational affiliation and setting
1	P4	Occupational therapist	Municipal healthcare; municipal size < 4000 inhabitants
1	P5	Physiotherapist	Municipal healthcare; municipal size < 4000 inhabitants
1	P6	Occupational therapist	Regional ambulatory rehabilitation team (ART) organised within specialist healthcare, supporting transitions, coordinating care, and guiding municipal service providers
1	P7	Nurse	Cross-sectoral team comprising municipal and hospital staff, coordinating transitions between healthcare levels primarily for elderly individuals
1	P8	Counsellor	Municipal Labour and Welfare Administration (Nav) municipal size > 50,000 inhabitants
2	P9	Occupational therapist	Municipal healthcare; municipal size > 50,000 inhabitants
2	P10	Physiotherapist	Municipal healthcare; municipal size > 50,000 inhabitants
2	P11	Occupational therapist	Regional ambulatory rehabilitation team (ART) organised within specialist healthcare, supporting transitions, coordinating care, and guiding municipal service providers
2	P12	Physiotherapist	Cross-sectoral team comprising municipal and hospital staff, coordinating transitions between healthcare levels primarily for elderly individuals
2	P13	Employment reintegration specialist	Municipal Labour and Welfare Administration (Nav) municipal size > 50,000 inhabitants

### Data generation

Data for this study were generated during a full-day interactive workshop held at a conference hotel in March 2022. The workshop began with a plenary session in which researchers reported summaries from previous workshops. This introduction served to remind the participants of past discussions, inspire further reflection, and establish a shared frame of reference for the subsequent focus group sessions. The participants then engaged in focus group discussions, guided by discussion guides developed for each group (Additional file 1). Each of the focus group discussions lasted between 2 ½ and 3 h and was interrupted only by a plenary meeting and a lunch break. The workshop schedule is outlined in Table 3.

**Table 3:** Workshop schedule and overview

Duration (5 h 45 m total)	Activities
30 m	Plenary introduction by the researchers
15 m	Break
1 h 15 m	Focus groups
30 m	Plenary presentation of focus group discussions thus far
1 h	Lunch break
1 h 15 m	Focus groups continue
15 m	Break
45 m	Plenary workshop summary, feedback, and communication of further project plans from the researchers

Two researchers moderated each focus group, with one serving as the main moderator and the other taking notes and asking supplementary questions. All the researchers were trained in qualitative research and had prior experience in conducting focus groups.

The focus group sessions were designed to facilitate comprehensive discussions on team-based long-term rehabilitation. The participants were actively engaged in sharing their perspectives and insights, which helped cultivate a shared understanding of the challenges and opportunities associated with team-based approaches. To ensure depth in the discussions and maintain a critical approach, the participants were encouraged to not only propose solutions and new ideas but also to voice any scepticism or reservations. This approach fostered an atmosphere of open dialogue and mutual knowledge exchange, thereby facilitating the cocreation of insights through debate and interaction.

The focus group sessions and the plenary session between the focus group sessions were audio recorded, resulting in a total of 6 h and 10 min of recorded material. These audio recordings constituted the primary data for this study and were transcribed verbatim, deidentified, and reviewed for accuracy by the researcher team prior to detailed analysis.

### Data analysis

The data analysis was performed in accordance with Barbour’s [74] approach to analysing focus group data. This approach involves a predominantly inductive, data-driven strategy for thematic analysis, involving constant comparison within and between focus groups. It also emphasises the broader context of focus groups during data interpretation and highlights the importance of researchers remaining reflexive throughout the analysis.

A key question guiding our initial analysis was ‘How do participants conceptualise the organisation and utilisation of teams in enhancing long-term rehabilitation for iwABI within the rural context of North Norway?’ Later in the process, we focused on more specific questions, such as ‘What challenges and opportunities do participants identify for implementing team-based approaches?’ and ‘How do participants envision collaboration between teams across different healthcare service levels?’ As the analysis progressed, we integrated insights from previous research to deepen our interpretation, drawing on the literature discussing healthcare teams [75–81], community integration after ABI [36, 81–84], and the need for a shift from impairment-focused to reengagement-oriented objectives during ABI rehabilitation trajectories [33, 51, 85].

The authors have expertise across the fields of occupational therapy (AG, CA), physiotherapy (LF, ME, MN), and sociology (HKA) as well as substantial experience in clinical rehabilitation and healthcare service research. MN primarily conducted the analysis, but the coauthors were involved in collaborative meetings, which facilitated continuous discussion during the analysis and drafting of the manuscript. This collective approach was crucial for improving the accuracy of interpretations, reinforcing research credibility, and encouraging ongoing reflexivity throughout the process.

### Ethical considerations

This research followed the principles outlined in the Declaration of Helsinki. The project was approved by the Norwegian Centre for Research Data (reference number 659996) and the Regional Committees for Medical and Health Research Ethics in Norway (reference number 237955). Before inclusion, participants received oral and written information about the project and provided signed informed consent. The participants were rigorously anonymised in the data presentation to ensure confidentiality and privacy.

## Results

**Fig. 3:**
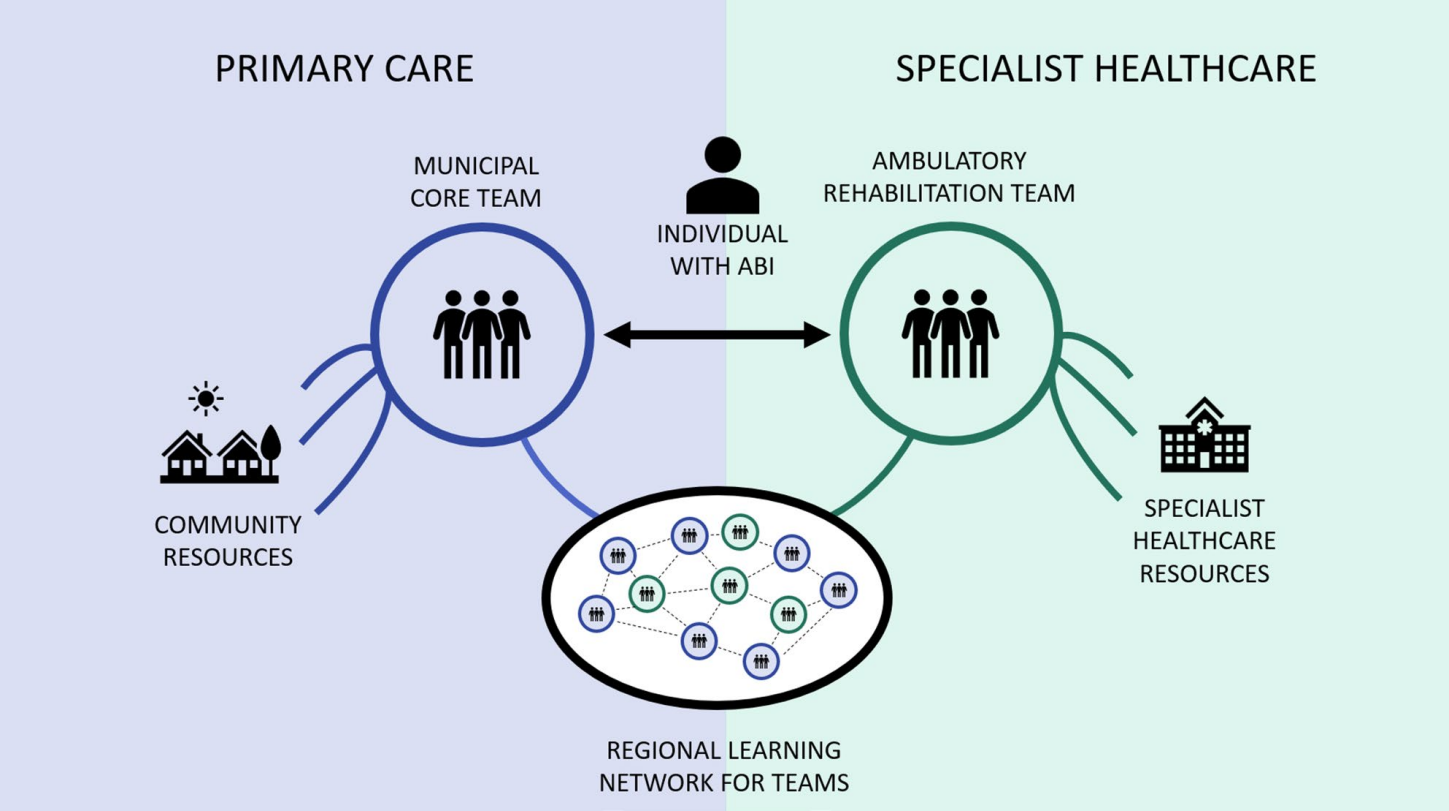
Illustration of a framework for team-based long-term ABI rehabilitation in rural North Norway

Our analysis identified four main themes related to enhancing team-based long-term ABI rehabilitation in North Norway (Table 4). First, participants advocated for the establishment of municipal ‘core teams’ to strengthen rehabilitation services and leverage local knowledge with the aim of enhancing the integration of iwABI into the community. Second, linking the municipal core teams with the existing ARTs was suggested to ensure care continuity and integrate specialised ABI expertise with local knowledge. Third, the participants underscored the potential benefits of ongoing collaboration between the core teams and the ARTs in improving the management of ABI rehabilitation trajectories. Fourth, teams were envisioned as platforms for professional growth, thereby promoting expertise development through intrateam learning and through the establishment of an interteam network for knowledge sharing among similar teams within North Norway. These four themes informed the development of a framework (Fig. 3) that is intended to guide the future application of team-based, long-term ABI rehabilitation services in North Norway and other regions with similar rural challenges.

In the results presentation, participants providing illustrative quotes are identified via participant IDs (e.g., P1, P2), as detailed in Tables 1 and 2.

**Table 4:** Overview of the main themes and subthemes

Main themes	Subthemes
Establishing municipal core teams for rehabilitation	(i) Core teams increase service resilience (ii) Core team organisation must be adapted to local conditions (iii) Core teams leverage local community knowledge
Team collaboration across healthcare levels	(i) Municipal core teams and specialist teams collaborate to enhance care continuity across healthcare levels (ii) Team collaboration facilitates integration of specialist healthcare knowledge and local community resources (iii) Local provider involvement enables face-to-face support
Integrating rehabilitation trajectory management into team practices	(i) Teams’ joint trajectory management obviates the need for external coordinators (ii) A designated contact person within each team ensures a consistent point of contact for iwABI and collaborating teams (iii) Prolonged involvement in ABI rehabilitation challenges teams to interact with the wider community
Fostering expertise through teamwork	(i) Team structures contribute to the development and retention of expertise (ii) Refined task distribution among municipal and specialist teams cultivates diverse skillsets (iii) A learning network supports education and knowledge sharing among similar teams within the region

### Establishing municipal core teams for rehabilitation

Both focus groups reached a consensus regarding the need to establish permanent ‘core teams’ within municipalities to enhance long-term rehabilitation for iwABI. This recommendation derived from the recognition that municipal HCPs often work in isolation, without structured collaboration with other service providers within municipalities. This isolation was seen as a significant threat to care continuity in a context with few professional care providers and high personnel turnover, which participants considered common characteristics of rural healthcare services. A key argument for the shift to team-based organisation within municipalities was to provide a more collaborative environment and make services more resilient to disruptions from staff changes:

*Municipal occupational therapist (P4)*: I have settled on the need for [municipal] teams. To avoid having all tasks loaded onto a single person and avoid vulnerability, among other things. The reality is that people quit their jobs from time to time, and then there are replacements.

*Researcher (MN)*: So, you’re imagining teams, right?

*Municipal occupational therapist (P4)*: I imagine teams! To create a service that is not so vulnerable.

Some participants questioned the feasibility of establishing permanent teams in rural municipalities due to the low number of HCPs and lack of staff stability. For example, several ART members experienced struggles in establishing contact with municipal services. One ART member said:

In the end, I was simply unable to get an *answer* [from anyone in the municipality]. But I called once more: ‘No, she’s quit her job. Now *I’m* responsible.’ There’s no continuity at all. That’s the reality. It’s so vulnerable in most of the municipalities that we’re supposed to support. So, how can you build a *team* when you can’t even find a single person to contact? (P6)

However, this line of argument was countered by several participants, who argued that team-based organisation should be seen as the solution to challenges with staff availability and care continuity, as illustrated by the following exchange:

*Participant with ABI (P2)*: In those cases, it is even *more* important to have a team. Because then it’s not so dependent on individuals. [With a team], there will be several people who pick up on things, not just one person. So, if there is one person who is doing a poor job, then you might have five others who are doing a darn good one.

*Researcher (LF)*: Yes. Or who are not on sick leave or maternity leave, or….

*Participant with ABI (P2)*: Yes. Exactly.

Another major point of discussion was the large variation in service organisation across municipalities, which is influenced by differences in geographical features, population size, and financial situations. Consequently, focus group participants found it challenging to agree on a uniform organisation and composition of teams that can be applied to all municipalities. Instead, they emphasised the need for a pragmatic approach where concrete team-based solutions are tailored to local conditions, such as the characteristics of available staff and the location of existing services.

Both focus groups highlighted municipal teams’ local knowledge and proximity to iwABI over time as key advantages that they can leverage to promote community integration and self-management. For example, participants anticipated that the municipal core teams would effectively stay up to date on the everyday lives of iwABI, thereby increasing the likelihood of discovering emerging needs, which participants considered an essential prerequisite for adequate support provision.

### Team collaboration across healthcare levels

The participants recognised the need for municipal staff to develop ABI rehabilitation expertise but also underlined the limited opportunity for specialisation in rural healthcare settings. To mitigate this issue, a key suggestion emerging from the focus group discussions was to create structured collaboration between the proposed municipal core teams and ABI experts within specialist healthcare, notably the ARTs already established in North Norway. This collaborative relationship was suggested to narrow the existing gap between primary and specialist healthcare providers. The gap was illustrated by ART members’ difficulties in engaging with local providers, thereby compromising the ability of the ARTs to assist with transitions from the hospital to the community, coordinate care, and offer guidance to municipal colleagues. The participants anticipated that utilising the core teams as consistent points of contact in the municipalities and fostering ongoing collaboration between them and the ARTs would reduce communication barriers and enhance continuity of care across healthcare levels. Collaboration between these teams was suggested not only to facilitate ongoing access to resources within specialist healthcare but also to integrate existing local community resources into the rehabilitation trajectory.

The participants envisioned that enhanced team collaboration would facilitate a more systematic transfer of care responsibility between the specialist and the municipal healthcare levels. This approach contrasts with the current, often haphazard transfer of responsibilities and inconsistent follow-up for iwABI after hospital discharge. However, the participants also acknowledged the need for a flexible approach to each handover, recognising the variability in needs among iwABI and differences in municipal organisation and resources. These points were discussed during the plenary session:

*Researcher (ME)*: Did you discuss the division of responsibilities between the two teams? When is the municipality responsible, and when is…?

*ART member (P11)*: No, because with the kind of patients that we are discussing now, there’s a need for a seamless transition rather than a stop and a restart. (…) Ideally, the ARTs assume a position where we’re not the ones doing the primary follow-up but rather form a part of a larger machinery.

*Researcher (ME)*: So, there are different layers?

*Municipal physiotherapist 1 (P12)*: Yes, and patient needs also vary a lot.

*Municipal physiotherapist 2 (P10)*: In my opinion, each patient’s needs should determine how long we work in parallel – how long the ARTs are with us before the municipality assumes full responsibility. It depends on what *expertise* is needed in each case.

The participants who had prior experience interacting with the ARTs recognised them as a vital source of ABI rehabilitation expertise within the region of North Norway. However, ART members cautioned against viewing ARTs as a one-stop solution for all challenges in postdischarge ABI rehabilitation. They emphasised the importance of involving municipal providers with local knowledge and the opportunity to offer in-person support. Although ARTs periodically travel to meet iwABI and service providers, their capacity for daily support and in-depth local community knowledge is limited. One ART member who worked on a team responsible for a vast region commented, ‘You know, we’ve got [that whole geographical area] to cover. And maybe the area is too big? Perhaps it should rather be split into three zones, for instance? But either way, the important thing for us is the local points of contact, those professionals.’ (P6).

### Integrating rehabilitation trajectory management into team practices

Shortcomings in service coordination emerged as a major concern during focus group discussions. Both service providers and iwABI lamented the poor implementation of individual care plans and care coordinators, which are legally mandated in Norway. However, the discussions in the focus groups revealed challenges extending beyond mere planning and coordination deficiencies. The participants also emphasised a more fundamental need to develop services aligning with the long-term needs of iwABI, which was succinctly highlighted by a participant: ‘Coordinators must have someone to coordinate! I mean, if one is to coordinate, there must be *somebody* to coordinate.’ (P7).

Several participants noted that the current model for service coordination leaves coordinators without the intimate knowledge required for individual care customisation. An occupational therapist working as a municipal coordinator expressed concerns with the gap between administrative roles and direct service provision during the plenary session:

I can read reports and handle administrative tasks, but my familiarity with patients isn’t as profound as those who provide direct services. (…) I’m capable of leading meetings and organising things, but what I can’t do effectively is to tailor for individual patients. I lack intimate knowledge of specific cases. So that’s the big downside of today’s system.^1^

The identification of these shortcomings sparked discussions on the benefits of integrating the management of individual rehabilitation trajectories into teams’ collaborative practices, potentially eliminating the need for external coordinators. This concept was articulated clearly during the plenary session, when a focus group responded to critical questions about whether their proposed reorganisation met existing legal requirements for coordinators. In defence of the group’s vision, a physiotherapist participant replied:

I think that we’re all quite constrained by the existing system. And with the current situation, we agree: It’s challenging for patients to be without standalone coordinators. However, we envisioned a more streamlined process where such a coordinator simply wouldn’t be necessary because the individual would be taken care of by these teams – first, one connected from the beginning, and then another taking over the responsibility in the municipality. (P12)

All participants with ABI emphasised the importance of having a consistent contact point within the healthcare system – ideally a person they already knew and trusted. In response, participants proposed the assignment of a contact person within each team while also highlighting the need to clearly define which team holds primary responsibility for follow-up at any given time to avoid confusion. This suggestion aimed not only to establish a consistent and familiar contact point for iwABI but also to provide contact points between the envisioned municipal core teams and specialist teams to facilitate ongoing collaboration and smooth care transitions.

Both focus groups recognised that the joint management of rehabilitation trajectories between municipal core teams and specialist teams could integrate knowledge about iwABI acquired during hospitalisation with a much-needed reassessment of their situation after community re-entry. This concept was articulated by a municipally employed physiotherapist during the plenary session:

The initial assessment can happen within specialist healthcare, but a lot will also be discovered after the patient is back home. Therefore, we need to get a system in place to uncover these things. We cannot rely solely on the evaluations made during hospitalisation or the findings at discharge as the basis for ongoing support. (P10)

Discussions on teams’ long-term involvement in ABI rehabilitation prompted a debate on the need for professional roles to adapt to evolving needs in iwABI. The participants noted that it often takes substantial time for iwABI to capture the full extent of the consequences of brain injuries. These injuries often impact the everyday lives of iwABI in profound ways, with issues ranging from losing driving privileges to difficulties interacting socially and maintaining relationships. Accordingly, participants agreed on the need for rehabilitation service models that facilitate HCPs’ ability to monitor and respond to a broad range of evolving needs.

However, the participants also discussed the possibility of reducing the need for professional involvement over time by promoting self-management and community integration. This led to reflections on issues such as HCPs’ involvement in return to work and social participation, the potential benefits of involving peers and nonprofit organisations, and when to phase out professional support. Several participants noted that consideration of these elements broadened their understanding of ABI rehabilitation and triggered reflection on the responsibilities of healthcare services versus the responsibilities of the wider community:

*ART member (P11)*: I think it is crucial to ask: What do [individuals with ABI] require to participate in life, and not just in work settings, but beyond? (…) They need someone who asks questions such as: What has happened? Who are you now? What things are important to you? And then helps them act on those insights.

*Participant with ABI 1 (P3)*: But who are supposed to do that? (…) I’m not quite sure whether it’s the job of professional providers to manage all aspects of it.

*ART member (P11)*: No, perhaps not entirely.

*Participant with ABI 2 (P2)*: But professionals might help us reach the point where we’re able to achieve progress ourselves. For instance, being able to explain to friends how our lives have changed.

*Participant with ABI 1 (P3)*: M-hm. That might be.

### Fostering expertise through teamwork

The focus group participants repeatedly emphasised the need for rehabilitation service professionals to possess expertise that aligns with the long-term needs of iwABI. A municipal physiotherapist highlighted the importance of enhancing ABI knowledge at the primary healthcare level:

This development will produce professionals who are not only knowledgeable but also genuinely curious about patients and able to move beyond the limitations of the current system. It’s about being profoundly interested in what it takes to help patients progress. (P10)

However, it was noted that the expertise gained by HCPs in municipalities settings often dissipates, underscoring the need for a framework that supports a more persistent growth of expertise among municipal providers. An ART member said:

We provide a lot of education and guidance in the municipalities, but it’s almost exclusively connected to isolated patient cases, and the expertise that is obtained by the service providers disappears after a while. Therefore, when the next case comes up, we must do the same thing all over again. (…) In my opinion, many municipal service providers are *very* skilled, but they lack the time and framework needed to produce a permanent increase in expertise. (P11)

The participants suggested that ongoing collaboration between the core teams and the ARTs could serve as a platform for building expertise through the handling of concrete cases. The establishment of municipal core teams would enable the ARTs to shift their efforts from locating and coordinating municipal providers to guiding and collaborating with their municipal colleagues. This shift would enhance the role of ARTs in developing competencies within municipal teams while enabling the ARTs to continue being directly involved in the care of iwABI.

Participants with ABI emphasised that their brain injuries profoundly affected their lives across multiple domains, leading to diverse needs that continued to evolve after hospital discharge. This underscores the importance of professional support extending beyond medical care and physical recovery. HCPs in the focus groups acknowledged the longevity and nonlinearity of the rehabilitation process after ABI, but also noted that many issues that are important for iwABI do not fall under the current purview of the professional care providers in the municipalities. The subsequent discussions indicated a need to clarify who is responsible for providing the broader, longer-term support that iwABI require. Thus, participants introduced the idea of municipal core teams cultivating competencies that complemented rather than replicated specialist healthcare. On several occasions, this was referred to as ‘generalist competence.’ A nurse participant with previous work experience from both specialist and primary healthcare commented:

The expertise within the municipality should complement what’s available in hospitals, also because hospitals lack certain types of expertise. Simply duplicating what the ARTs do is not the solution. (…) [T]here is a need for specialised expertise somewhere in the system, while in other areas, it needs to be broader and more generalised. (P7)

To further amplify the benefits of team-based structures, participants proposed the formation of a learning network across North Norway. This network was envisioned to concentrate on professional education and knowledge sharing across several municipal teams and ARTs, thus enhancing the overall quality of services through sustained expertise development. An ART member reflected on the possible impact of such a network:

You can make as many laws and regulations as you like, but the reality is that the quality of the services ultimately hinges on the expertise of individual providers. (…) If we’re supposed to establish teams all around, they’re going to need expertise. So, having a network to foster expertise development is going to be crucial. (P6)

## Discussion

This study explored how team-based organisation can enhance long-term rehabilitation services for iwABI in rural North Norway. Our analysis identified four key themes: (1) establishing municipal ‘core teams’ to strengthen local rehabilitation services; (2) fostering ongoing collaboration between the core teams and existing specialist healthcare teams; (3) sharing management of rehabilitation trajectories between the teams; and (4) utilising teams to develop and retain expertise. These themes have guided the development of a framework (Fig. 3) designed to support the application of team-based approaches to long-term ABI rehabilitation. This framework assumes the presence or creation of specialist rehabilitation teams such as ARTs in North Norway. The following sections expand on the proposed framework and highlight key conditions for its success.

### The importance of stable team membership

Although Norwegian authorities emphasise team-based care for individuals with complex long-term needs [42, 50, 62], recent initiatives to implement this care in municipal settings have encountered substantial challenges [86–88]. A key issue is the reliance on standalone coordinators tasked with bridging fragmented services, often building collaboration across disciplines for each new case [86, 87]. While these ad hoc groups are flexible, they do not meet the necessary criteria for effective rehabilitation teams [58, 75].

In contrast, the municipal ‘core teams’ proposed in this study offer consistent team membership, which aligns better with effective team models in the literature [77, 89, 90]. Stable team membership fosters mutual familiarity and a collective dedication to the team’s success, which subsequently enhances communication, collaboration, and coordination within teams [76, 90, 91]. These factors are pivotal in improving care continuity [92], thus underscoring the importance of stable team membership.

### Adaptive teams for rural settings

Our results highlight that achieving team stability in rural areas can be challenging. Geographic expansiveness, scarcity of HCPs, and a broad scope of practice often require HCPs in rural primary healthcare to fulfil diverse roles and tasks [93]. Resource allocation to teamwork can detract from other important tasks. These conditions necessitate a flexible approach to team organisation in rural settings to balance the demands on HCPs.

The concept of ‘adaptive teams’ is particularly relevant in this context [77]. It emphasises aligning team organisation with the team’s intended purposes and the local conditions in which it operates rather than adhering strictly to rigid conceptualisations of teamwork. Adaptive teams are characterised not only by integrating members closely but also by adjusting their work to the specific context and the needs of the individuals they serve. This approach recognises that while tightly integrated teamwork is valuable, not every situation requires collective team action. It allows for flexible involvement, where team members may independently address issues or engage in less integrated forms of interprofessional work, such as collaboration, coordination, or networking [77, 78].

Integrating this pragmatic approach into our framework can help balance rural challenges, thus offering the necessary flexibility for stable yet responsive teams. While perfect team stability may be unattainable, effective teamwork can still be maintained through dynamic adjustment and flexible collaboration.

### Shifting from prescriptive to pragmatic team composition

Within the field of rehabilitation, teams are valued for integrating diverse professional perspectives and offering coordinated care [94–96]. However, the results of this study did not feature discussions on team composition. This may reflect an implicit assumption of standard disciplinary representation as required by Norway’s legal staffing requirements in municipalities, which mandates the inclusion of professional groups such as physiotherapists, occupational therapists, and nurses [97]. However, our findings indicate that the long-term needs of iwABI often extend beyond the current scope of practice for professionals in municipalities in North Norway. Therefore, simply adopting a service model in which a variety of professions are represented may not be sufficient to address the complexities of long-term ABI rehabilitation.

Some authors argue that the distinct contributions of each discipline are less important as iwABI progresses through the rehabilitation trajectory, with attention increasingly shifting towards reintegration into the community and the return to valued roles and activities [63, 91]. The authors suggest that softening traditional disciplinary boundaries can foster greater flexibility and adaptability among team members, prioritising a unified team vision over strict adherence to a predefined multidisciplinary composition [63, 91]. This pragmatic approach may be particularly relevant in rural settings, where the availability and diversity of HCPs are limited. However, further discussion on aligning team objectives with the long-term needs of iwABI is needed.

### Aligning team objectives with long-term needs after ABI

Team efficacy hinges on a clear and shared understanding among team members about the challenges they are meant to address [76, 98, 99]. On the other hand, ambiguous objectives are known to undermine team performance [90, 98]. Therefore, clear objectives are essential for effective teams in long-term ABI rehabilitation.

Recent studies highlight the need for a shift in rehabilitation objectives when iwABI transition from inpatient to community settings [33, 85]. Rather than focusing predominantly on impairment correction (i.e., restorative approaches), several authors propose that long-term rehabilitation should increasingly support iwABI in understanding their condition, fostering self-management, and adapting to living with long-term impairments (i.e., adaptive approaches) [51, 84, 85, 100]. This shift implies a person-centred approach that extends beyond physical recovery to include social, psychological, and emotional support as well as the promotion of community integration. This shift will influence the overarching objectives of teams involved in long-term ABI rehabilitation.

After ABI, residual impairments are common and typically stabilise within three to six months. However, substantial evidence shows that iwABI can have fulfilling lives despite persistent cognitive and motor impairments [101–103]. Therefore, rehabilitation objectives that emphasise everyday life activities and community integration become increasingly relevant for long-term rehabilitation success [33]. Such objectives are particularly appropriate in community settings, where rehabilitation processes have no definite time limit and individuals’ needs and goals become more diverse, personal, and context dependent [84, 85]. Aligning team objectives with these broader, person-centred goals ensures that long-term ABI rehabilitation effectively addresses the evolving needs of iwABI.

### Collaboration across healthcare levels to integrate resources and address rural challenges

The collaboration between primary and specialist healthcare teams proposed in this study aligns with the framework outlined by Wade [81]. Wade’s framework highlights the benefits of ongoing collaboration between teams specialising in specific conditions and teams providing long-term care in community settings. The framework also emphasises the crucial role of community-based teams in facilitating social participation and community integration due to their proximity to individuals’ daily lives [81]. Our study extends the previous framework by suggesting that ongoing collaboration between teams across healthcare levels can effectively address rural-specific challenges such as geographical distances, low population density, and a broad scope of practice for HCPs.

Our findings also emphasise the need for collaborating teams’ competencies to complement rather than duplicate each other. Wade [81] addresses this issue by questioning the traditional distinction between ‘community’ and ‘specialised’ services, suggesting that ‘specialised’ services could encompass a broader range of services, including teams specialising in rehabilitation within community settings. This perspective is echoed in the current study, thus highlighting the need for a nuanced discussion about the expertise required for effective long-term ABI rehabilitation. Specifically, our framework suggests that a refined distribution of tasks and expertise between primary and specialist healthcare teams can benefit iwABI in rural areas such as North Norway by ensuring that services remain responsive and adaptable over time.

A challenge for specialist outreach teams such as ARTs is that, while they provide disease-specific expertise, excessive dependence on them can hinder skill development in primary healthcare [104]. To address this, our framework emphasises ongoing collaboration between core teams and ARTs. This approach not only facilitates access to ABI-specific expertise but also promotes professional development and skill retention within the municipal workforce. Conversely, municipal core teams must balance the development of ABI-specific expertise with addressing the diverse needs of populations in rural communities. Previous studies in Norway have indicated that adopting disease-specific specialisation in primary healthcare can lead to service fragmentation and poor resource utilisation [105–107]. Our framework aims to integrate local knowledge with ABI-specific expertise, thereby fostering adaptive and comprehensive long-term care. While the proposed structured and sustained collaboration between primary and specialist healthcare teams with complementary areas of expertise may not fully resolve the tension between specialisation and flexibility, this approach can at least help mitigate service fragmentation and enhance resource utilisation.

### Potential role of teams in enhancing collaboration between communities and services

Although rehabilitation processes often span several years for iwABI, the degree of professional support tends to decrease rapidly after discharge [14, 15, 21, 24, 108, 109]. This has led to calls for service models that address the evolving long-term needs of iwABI [30, 110–114]. This study highlights the potential role of collaborating healthcare teams in ‘reassessing the situation’ after iwABI return to their communities. Our findings suggest that healthcare teams may need to expand their roles and responsibilities, thus potentially broadening the scope of rehabilitation services and prolonging service involvement to better address the evolving long-term needs of iwABI.

Our framework extends beyond the contributions of professionals and formal rehabilitation services by highlighting the role of municipal core teams in fostering synergies between professional services and community resources. This aspect of the framework addresses recent critiques that the discourse on service delivery models often prioritise professional perspectives and overlooks the substantial contributions to long-term care made by nonprofessionals in the community [82, 83]. Integrating informal caregivers and other community resources alongside professional providers is suggested in order to reduce care fragmentation and increase care capacity by leveraging existing community strengths [82, 83]. These considerations resonate with a recent study highlighting the advantages of developing inclusive communities and rural ABI rehabilitation services together to enhance community care capacity and drive relevant service improvements in rural areas simultaneously [115]. These perspectives can inform future efforts to adopt and apply the framework outlined in this study.

### Innovating without increasing organisational complexity

To date, rehabilitation services have developed in a piecemeal rather than a preplanned way, resulting in a complex and fragmented system that lacks a coherent underlying organisational model [58, 63]. Recent Norwegian studies have shown that introducing new organisational elements into the existing rehabilitation service system, such as intermunicipal teams [116] or independent care coordinators [117], adds complexity and creates unexpected coordination challenges. Our framework addresses these issues by predominantly avoiding the introduction of additional elements, building on existing municipal resources to form core teams. While ARTs already exist in North Norway, other regions may need to establish similar specialist teams to provide the necessary foundation for the framework proposed in this study.

By building on existing infrastructure, our approach offers an innovative yet pragmatic way to reduce service fragmentation and enhance care continuity. This strategy is supported by previous studies on service integration between primary and specialist healthcare, which suggest that organisational merging is not a prerequisite for achieving person-centred care [118, 119]. These studies indicated that enhancing interservice collaboration through partnerships and networks is more effective than comprehensive organisational changes. These previous studies also emphasised the importance of appraising possibilities within the existing system and coproducing new care models with all stakeholders [118, 119], which aligns with our study’s methodology.

### Team learning through rehabilitation trajectory management

Our framework highlights the dual roles of teams in managing rehabilitation trajectories and serving as platforms for expertise development. Other authors have emphasised the interplay between these aspects, proposing that managing complex cases provides valuable learning opportunities for teams [79, 80]. According to Stabell & Fjeldstad [80], teams’ unique capacity to address complex issues is not limited to problem solving but also involves prior problem formulation and subsequent evaluation of actions. The iterative cycle of problem formulation, problem solving, and evaluation inherently involves learning. In other words, tailoring solutions for individual cases can also enhance teams’ overall ability to address future challenges [80]. Similarly, Sandberg [79] highlights that high-performing teams are characterised by continuous improvement and expansion of their skill sets, noting that failing to leverage learning opportunities represents a significant waste.

These insights support the idea of teams as platforms for expertise development and underline the integrated nature of teams’ clinical work and learning processes. They also reinforce our framework by underlining the importance of learning processes both within and across teams. This learning can occur through day-to-day collaboration between municipal core teams and specialist teams, as well as through learning networks among teams across the region, as illustrated in Fig. 3. The previous successful application of models for knowledge sharing and continuous learning in other rural areas, such as virtual communities of practice [120], further illustrates the potential for this approach. Overall, the combination of learning within and across teams aims not only to enhance the management of individual rehabilitation trajectories but also to provide a sustainable model for expertise development, thereby ensuring that teams remain adaptive and proficient in addressing the evolving long-term needs of iwABI.

### Limitations

This study utilised qualitative data from focus groups, which offered rich insights but may not encompass the full range of experiences and opinions among stakeholders in ABI rehabilitation. Furthermore, the unique healthcare challenges and organisational structure of rural North Norway may limit the applicability of our findings to other regions or urban environments.

The composition of our focus groups, with a greater proportion of professionals compared with iwABI, was intended to capture a wide range of professional experiences across different phases of the rehabilitation trajectory. However, this approach may have limited the representation of iwABI perspectives. Additionally, the absence of leaders and policymakers from the discussions may have resulted in an incomplete exploration of systemic, organisational, and policy-related factors. Future studies should consider incorporating these viewpoints to provide a more comprehensive evaluation of the proposed framework for team-based rehabilitation services.

## Conclusions

This study highlights how team-based organisation can enhance long-term rehabilitation services for iwABI within the rural context of North Norway. Based on our findings, we propose a framework centred around the establishment of municipal ‘core teams’ at the primary healthcare level and fostering their collaboration with existing specialist healthcare teams. The key to this framework is the ongoing collaboration across healthcare levels and the joint management of rehabilitation trajectories, which ensures that teams remain adaptive, proficient, and responsive to the evolving long-term needs of iwABI. Our framework is designed to promote sustained professional support and community integration of iwABI as well as the development and retention of expertise among HCPs. While the framework is tailored to the specific context of rural North Norway, the underlying principles of structured collaboration and adaptive care could also be beneficial in other regions. Future research should examine the application and evaluation of this framework to assess its impact on outcomes for iwABI, professional development, and overall service effectiveness.

## Supplementary Information

Below is the link to the electronic supplementary material.

Supplementary Material 1

## Data Availability

The datasets generated and analysed in the current study were conducted specifically for this research, and findings based on these data have not been previously published. Due to the sensitive nature of data and to protect participant confidentiality, the full transcripts are not publicly available. However, anonymised excerpts supporting the findings are included within the article. Additional de-identified data are available from the lead researcher and coauthor (CA) upon reasonable request. Data are securely stored in a controlled access data repository at UiT The Arctic University of Norway.

## Footnotes

1 This participant did not take part in either of the focus groups referred to in Tables 1 and 2 but made the comment during Focus Group 1’s presentation in the workshop’s plenary session.

## Abbreviations

ABI: Acquired brain injury
ART: Regional Ambulatory Rehabilitation Team organised at the specialist healthcare level
EBCD: Experience-based Co-design
HCPs: Healthcare professionals
iwABI: Individuals with ABI
TBI: Traumatic brain injury
