# Supplementary material for: ‘I imagine teams!’ – exploring the potential of team-based long-term brain injury rehabilitation in North Norway
Source: BMC Health Serv Res. 2025 Dec 29;26:15. doi: 10.1186/s12913-025-13894-0 (PMC12772063; doi:10.1186/s12913-025-13894-0)
Supplement: Supplementary file 1 — Supplementary Material 1 [file 12913_2025_13894_MOESM1_ESM.docx]

# Focus group discussions guide (Workshop 3)

Group code H1 (Nickname: “Team, rural areas”)

**Moderators:** MN (main moderator), AB

**Session schedule:** 09.15–10.30 (Session 1), 12.30–13.45 (Session 2)

**Available equipment:** Large map of North Norway; paperboard cards with illustrations of diverse stakeholders, teams, and arenas; sticky notes; drawing paper; pens; permanent markers

## Introduction (10 minutes)

- Brief introductory round with the participants and moderators.
- Quick round of spontaneous reactions to the plenary introductory session.

## Presentation of experiences with team-based work in North Norway (15 minutes)

Two focus group participants present their experiences with working in teams:

- Experience of working in an Ambulatory Rehabilitation Team (ART).
- Experience of working in a cross-sectoral team coordinating transitions between healthcare levels.

## Presentation of the group’s assignment (5 minutes)

Main moderator (MN) presents:

- The group’s specific assignment for the session.
- Outline of the schedule and plan for today’s work.
- A reminder of the questions sent to the focus group participants via email before the workshop.

## Discussion on approaches for teams and/or coordinators in North Norway (60 minutes)

### Topic 1: Solutions adapted to rural areas

- Opening question: *“If the overarching goal of rehabilitation for individuals with ABI is to promote a good everyday life and community integration: How can services be organised to achieve this? What role might team-based approaches play in this?”*
  - What expertise is needed?
  - What are the roles of teams in specialist vs. municipal healthcare?
  - How can teams help “activate local resources”?

### Topic 2: Team solutions

- Is one team sufficient, or are multiple teams needed?
  - What type(s) of team(s) is/are needed?
  - Where should it/they be located?
  - With whom should the team(s) collaborate, and how?
  - Who should be part of the team(s)?
  - Which professions or disciplines should be involved?
  - Should the team(s) work across sectors or remain within a single sector?

### Topic 3: Team(s) as part of a larger rehabilitation service model

- What could the relationship be between the team(s) and the potential future “Power Centre” envisioned in RehabLos?
- What role could the use of web-based and digital solutions play for team coordination?

### Topic 4: Team’s underlying rationale and philosophy

- What are the potential consequences of focusing on everyday life and community integration?

### Topic 5: Team coordination and rehabilitation planning

- What necessitates service coordination today? What could reduce the need for coordination?
- Is “coordinator” the right term, or could another term be more fitting?
- Should coordinators be part of the team(s), or would it be better to have stand-alone coordinators?

### Individual task work (45 minutes)

1. 10 minutes of individual work.
2. Group discussion on how each participant approached the task.
3. Agreement on a proposal: Can the group agree on how teams and/or coordinators should function in long-term ABI rehabilitation in North Norway?

### Conclusion (15 minutes)

- What are the key priorities from today’s discussion?
- Is there anything important that we have not covered today? If so, now is the time to bring it up.

## Individual task

- Envision that the RehabLos project has received additional funding.
- A new model for rehabilitation will be piloted in a few municipalities.
- Teams and coordinators are part of the model.
- You have been hired as a consultant for the project and given the following task:

“Provide your best recommendations on what needs to be in place for a team and/or coordinator arrangement to work well for individuals living in *name of a small, rural municipality*.

Please read the vision for RehabLos before you start working on the task.

**
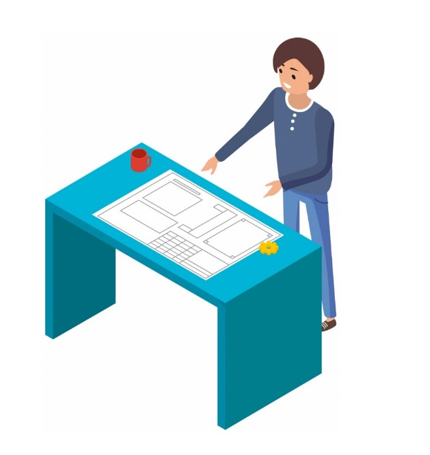
**Vision for RehabLos:

“All individuals of working age who suffer a brain injury and return home should be supported throughout their entire journey back to a meaningful everyday life and inclusion in society, regardless of where they live in North Norway.”

# Focus group discussions guide (Workshop 3)

Group code H2 (Nickname: “Team, community integration”)

**Moderators:** LF (main moderator), AG

**Session schedule:** 09.15–10.30 (Session 1), 12.30–13.45 (Session 2)

**Available equipment:** Drawing paper, pens

## Introduction

- Brief introductory round with the participants and moderators.

## Presentation of the group’s assignment

Main moderator (LF) presents:

- The group’s specific assignment for this session.
- Outline of the schedule and plan for today’s work.
- A reminder of the questions sent to the focus group participants via email before the workshop.

## Discussion on team-based approaches for community integration

### Topic 1: Professional roles and competencies

- Exploring the views of professionals. (Give each profession room to discuss their expertise.)
  - If we are going to work in teams: What is important?
  - Do you have previous experience with team-based work? What were the challenges or successes?
  - How would you prefer to work within a team?
  - What are the specific competencies and contributions of each profession? What do you consider your most important contribution to a team?
  - Do you perceive that your profession has the responsibility and competence for long-term ABI rehabilitation (i.e., “life rehabilitation”)?
- Participants with ABI perspectives:
  - What is the potential for collaboration between different professions and stakeholders from your perspective?

### Topic 2: Cross-sectoral collaboration

- How do we collaborate across sectors?
- What is the role of the specialist healthcare sector?
- What types of healthcare service integration do you envision?
- Consider the continuum of collaboration: fusion, network, or case-management? (Chain, web, or hub?)

### Topic 3: Roles and objectives in team(s)

- What should the job description for members of the team(s) should look like?
- When can we say that rehabilitation have been successful? What is the overarching goal?
- First consider: What is/are the overarching objective(s) of the team(s)? Then: What is/are the different role(s) within the team(s)?
- Work in pairs and then present for each other.

## Conclusion

- What are the key priorities from today’s discussion?
- Is there anything important that we have not covered today? If so, now is the time to bring it up.
